# Swedish snus use is associated with mortality: a pooled analysis of eight prospective studies

**DOI:** 10.1093/ije/dyaa197

**Published:** 2020-12-22

**Authors:** Marja Lisa Byhamre, Marzieh Araghi, Lars Alfredsson, Rino Bellocco, Gunnar Engström, Marie Eriksson, Maria Rosaria Galanti, Jan-Håkan Jansson, Anton Lager, Michael Lundberg, Per-Olof Östergren, Nancy L Pedersen, Ylva Trolle Lagerros, Weimin Ye, Patrik Wennberg, Cecilia Magnusson

**Affiliations:** 1 Department of Public Health and Clinical Medicine, Family Medicine, Umeå University, Umeå, Sweden; 2 Department of Public Health Sciences, Karolinska Institutet, Stockholm, Sweden; 3 Institute of Environmental Medicine, Karolinska Institutet, Stockholm, Sweden; 4 Department of Medical Epidemiology and Biostatistics, Karolinska Institutet, Stockholm, Sweden; 5 Department of Statistics and Quantitative Methods, University of Milano-Bicocca, Milan, Italy; 6 Department of Clinical Sciences, Lund University, Malmö, Sweden; 7 Department of Statistics, Umeå School of Business, Economics, and Statistics, Umeå University, Umeå, Sweden; 8 Centre for Epidemiology and Community Medicine, Region Stockholm, Stockholm, Sweden; 9 Department of Public Health and Clinical Medicine, Skellefteå Research Unit, Umeå University, Skellefteå, Sweden; 10 Social Medicine and Global Health, Department of Clinical Sciences, Lund University, Malmö, Sweden; 11 Department of Medicine, Clinical Epidemiology Unit, Karolinska Institutet, Stockholm, Sweden; 12 Center for Obesity, Academic Specialist Center, Stockholm Health Services, Stockholm, Sweden

**Keywords:** All-cause mortality, cancer mortality, cardiovascular mortality, moist oral snuff, smokeless tobacco, Swedish snus

## Abstract

**Background:**

The health consequences of the use of Swedish snus, including its relationship with mortality, have not been fully established. We investigated the relationship between snus use and all-cause and cause-specific mortality (death due to cardiovascular diseases, cancer diseases and all other reasons, respectively) in a nationwide collaborative pooling project.

**Methods:**

We followed 169 103 never-smoking men from eight Swedish cohort studies, recruited in 1978–2010. Shared frailty models with random effects at the study level were used in order to estimate adjusted hazard ratios (aHRs) and 95% confidence intervals (CIs) of mortality associated with snus use.

**Results:**

Exclusive current snus users had an increased risk of all-cause mortality (aHR 1.28, 95% CI 1.20–1.35), cardiovascular mortality (aHR 1.27, 95% CI 1.15–1.41) and other cause mortality (aHR 1.37, 95% CI 1.24–1.52) compared with never-users of tobacco. The risk of cancer mortality was also increased (aHR 1.12, 95% CI 1.00–1.26). These mortality risks increased with duration of snus use, but not with weekly amount.

**Conclusions:**

Snus use among men is associated with increased all-cause mortality, cardiovascular mortality, with death from other causes and possibly with increased cancer mortality.

**Key Messages**

- In this large pooling project including data from eight prospective studies, we systematically examined associations of snus use with mortality, overall and according to cause of death, among Swedish men.
- Snus use was associated with increased all-cause mortality, including mortality from cardiovascular disease and from causes other than cardiovascular disease and cancer. There was also an association with cancer mortality.
- Because of the widespread use of snus in the Nordic countries, its association with mortality, if causal, has important public health implications.
- Our findings may also be relevant for improving understanding of the health effects of other non-smoked tobacco and nicotine products such as e-cigarettes.

## Introduction

Swedish snus, a smokeless tobacco product, is a moist powder of fermented ground tobacco. A pinch or a portion-bag is placed under the lip, where the active constituents are absorbed through the oral mucosa.^1^ The highest prevalence of current snus use is found in Sweden, where 22% of men and 4% of women use it daily.^2^ Snus is also common in Norway and has a growing number of users in the USA.^3–5^

Snus contains a number of harmful substances, including heavy metals, polyaromatic hydrocarbons, tobacco-specific nitrosamines and tobacco alkaloids.^6^ Nicotine is the most abundant of the alkaloids, and has physiologic effects on the cardiovascular system,^7–10^ with potentially negative effects on human health. The addictive properties of nicotine frequently result in prolonged snus use,^1^ entailing long-term exposure to potential toxicants among users.

The snus-using population in Sweden provides a basis for solid research on non-smoked nicotine that is hard to find elsewhere. In addition to elucidating the health consequences of snus use, our research findings may also generate hypotheses regarding the safety of other nicotine products, for example nicotine replacement therapy and e-cigarettes.

Whereas the use of snus has been associated with adverse health outcomes such as obesity,^11^^,^^12^ type 2-diabetes,^13^ heart failure,^14^ and oesophageal and rectal cancer,^6^^,^^15^^,^^16^ the influence of snus use on all-cause mortality needs further attention. Two existing reports indicate an excess overall mortality risk from snus use, mainly due to increased cardiovascular mortality, but their interpretation is hampered by lack of control for important confounders.^17^^,^^18^

The Swedish Collaboration on Health Effects of Snus Use includes individual participant data from several Swedish prospective studies.^19–26^ The collaboration was established in order to clarify the impact of snus use on health across time and geographical regions, with adequate statistical power and control for confounding factors including smoking. Here, we use this pooling project to investigate the association between snus use and all-cause, cardiovascular, cancer and other cause (non-cardiovascular and non-cancer) mortality in men with no history of smoking.

## Methods

### Contributing studies and data collection

In all, 383 015 participants were derived from eight population-based cohorts from diverse geographic regions across Sweden (Table 1). The principal investigator of each cohort provided individual participant data, and data harmonization and analyses were implemented centrally. Details of study design and data collection procedures of the individual studies have been published elsewhere.^19–26^

**Table 1:** Characteristics of included cohorts in the Swedish collaboration of health effects of snus use

Study	Study population	Data collection	Period of recruitment	Study end	Male participants (*n*)	Person years of follow- up (*n*)	Mean age at recruit- ment (years)	Mean age at death (years)	Deaths (N)	Current smokers (%)	Current snus users (%)	Information available regarding snus use		
												Duration	Amount	Former use
Construction Worker Cohort (CWC)	All workers in the Swedish construction industry	Questionnaire	1978–1993	2004	279 897	5 777 263	34	66	31 429	46	27	Yes	Yes	Yes
Malmö diet and Cancer Study (MDCS)	Population-based, Malmö City	Questionnaire	1991–1996	2013	12 120	207 755	59	75	4372	27	7	No	Yes	No
Multinational Monitoring of Trends and Determinants in Cardiovascular Disease (MONICA)	Population-based, Norrbotten and Västerbotten Counties	Questionnaire	1986–2004	2008	4563	57 222	48	70	643	22	24	Yes	Yes	Yes
National March Cohort (NMC)	Participants in a charity walk, national	Questionnaire	1997	2010	15 318	193 423	52	78	2531	7	9	Yes	Yes	Yes
Scania Public Health Cohort (Scania_PHC)	Population-based, Scania County	Questionnaire	1999–2000	2008	6201	56 092	48	76	231	21	20	No	No	No
Screening Across the Lifespan Twin Study (SALT)	Twins born in Sweden between 1926 and 1958, national	Structured telephone interview	1998–2002	2010	18 331	177 243	56	71	2522	17	16	Yes	Yes	Yes
Stockholm Public Health Cohort (Sthlm_PHC)	Population-based, Stockholm County	Questionnaire	2002–2010	2011	39 406	188 704	50	76	1465	13	18	No	No	Yes
Work, Lipids and Fibrinogen Study (WOLF)	Employees in Väster-norrland, Jämtland and Stockholm Counties	Questionnaire	1992–1997	2009	7189	100 373	42	61	265	20	23	Yes	Yes	Yes
Total			1978–2010	2004–13	383 025	6 758 075	39	68	43 458	38	24			

**Figure 1:**
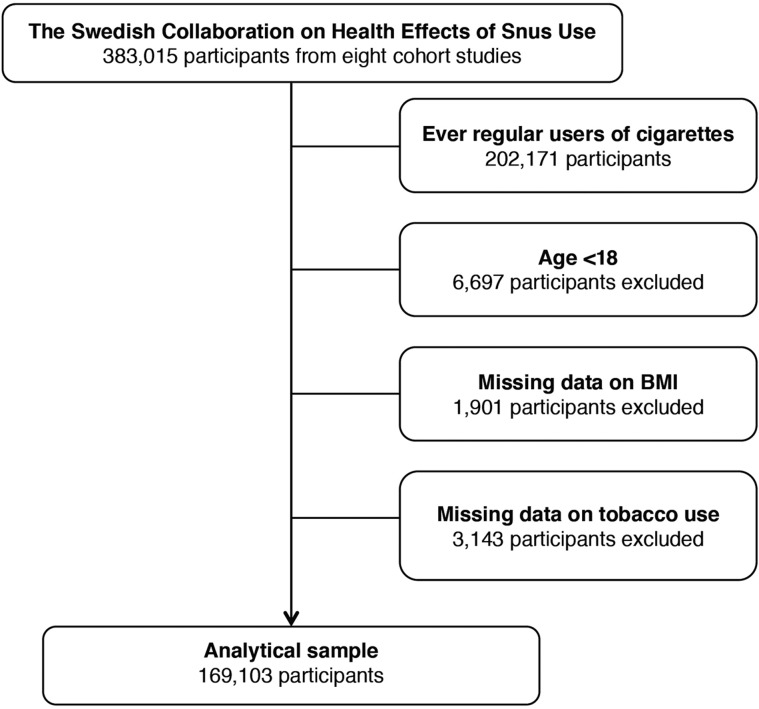
Exclusion of participants

Since snus use is rare in women, we restricted the study to men. To eliminate potential residual effects of current or previous cigarette smoking, we excluded all participants reporting ever regular use of cigarettes (*n* = 202 171). Additional exclusion criteria were age <18 years (*n* = 6697), missing information on body mass index (BMI) (*n* = 1901) and missing information on tobacco use (*n* = 3143). Thus, our main analyses included 169 103 never-smoking men (Figure 1).

We followed participants for cause-specific mortality by linkage with the National Cause of Death Register,^27^ from which we obtained the main cause of death according to the *International Classification of Diseases* (ICD). For overall mortality, we included deaths from all causes. For cardiovascular disease (CVD) mortality, we included deaths related to the heart and circulation system coded as 390–459 in ICD-9 (ICD, 9th Edition) and I00–I99 in ICD-10 (10th Edition). We defined cancer mortality as deaths due to malignant neoplasms, coded as 140–208 and C00–C97 according to ICD-9 and ICD-10, respectively. Other cause mortality was defined as deaths from all causes except CVD and cancer, and included e.g. mortality from respiratory diseases, infections, accidents and suicide.

Information on tobacco use was collected at baseline using self-administrated questionnaires in seven studies^19–23^^,^^25^^,^^26^ and by a structured phone interview in one study.^24^ All studies contributed information on self-reported current snus use, and six studies also provided data on former snus use^19^^,^^21^^,^^22^^,^^24–26^ and weekly amount of snus.^19–22^^,^^24^^,^^26^ Five studies contributed data on duration of snus use.^19^^,^^21^^,^^22^^,^^24^^,^^26^ Information on potential confounding factors that could be harmonized across the studies was collected when available (Table 2). Information on height and weight, whether it was self-reported or measured by health professionals, was available in all studies, whereas education level^28^^,^^29^ and alcohol consumption^30^^,^^31^ had been assessed in all studies except one.^19^ Data on physical activity^28^^,^^32^ were self-reported and retrieved from six studies.^21–26^

**Table 2:** Baseline characteristics of study participants in the pooled analytical sample

Characteristics	All	Never-users of tobacco	Exclusive current snus users	Exclusive former snus users
Participants (*n*)	169 103	124 256	39 156	5 691
Age, years (mean ± SD)	36 ± 15	38 ± 16	29 ± 11	38 ± 13
BMI, kg/m^2^ (mean ± SD)	24.5 ± 3.2	24.6 ± 3.2	24.1 ± 3.2	25.0 ± 3.1
Alcohol intake^a^^,^^b^							
Never (%)	12.21	14.06	3.85	4.08
Low (%)	31.45	33.06	22.72	27.39
Medium (%)	28.45	27.71	30.28	35.02
High (%)	22.98	20.18	37.91	30.43
Missing (%)	4.91	4.99	5.24	3.08
Educational level^a^							
≤9 years (%)	22.09	23.15	18.13	15.61
10–12 years (%)	40.83	38.65	52.58	46.37
≥13 years (%)	35.28	36.27	27.89	37.06
Missing (%)	1.80	1.92	1.39	0.97
Physical activity level^a^							
0–2 h of light activity per week (%)	9.74	9.47	12.40	8.02
>2 h of light activity per week (%)	32.92	32.35	37.30	31.69
1–2 h of exercise per week (%)	33.33	33.59	29.71	37.45
>2 h of exercise per week (%)	15.59	14.96	17.31	20.77
Missing (%)	8.41	9.63	3.28	2.26

a In cohorts that provided information on these covariates (alcohol intake^20–26^, educational level^20–26^, physical activity level^21–26^).

b Alcohol consumption here categorized into never, low, medium or high use, in tertiles of grams consumed per week.

The specific studies were approved by their respective regional ethical vetting boards, and approval for the collaboration was granted by the Stockholm Regional Ethical Review Board (registration number 2009/971-31/3).

### Statistical analysis

We categorized snus use into never, former and current use (where non-current snus use was treated as never use in the studies that did not have information on former snus use). Furthermore, we categorized current use according to amount consumed per week (<4 cans, 4–6 cans, ≥7 cans) and duration of use (<5 years, 5–9 years, 10–14 years, ≥15 years). Never-users of snus formed the reference group. Categorization, in both analyses, was based on available response options in the cohorts’ questionnaires.

Each individual contributed person-time from the date of entering into the respective cohort until the date of death, or the end of the cohort, whichever came first. We used gamma-distributed shared frailty models with random effects at the cohort level to estimate adjusted hazard ratios (aHRs) and corresponding 95% confidence intervals (CIs) of death in relation to snus use, using attained age (in years) as the time scale. The shared frailty model is an extension of the Cox proportional hazards model and accounts for within study correlation by incorporating shared random effects. In addition to the inherent adjustment for age, all models were adjusted for body mass index (BMI, in kg/m^2^). The underlying assumption of proportional hazards was tested using Schoenfeld’s global test.^33^

Statistical analyses were conducted using Stata statistical software (Version 13.0, Stata Corporation, and College Station, TX, USA).

### Sensitivity analyses

We conducted sensitivity analyses according to the following scenarios. (i) Excluding the Construction Workers Cohort, which was by far the largest cohort, hence possibly driving the results. (ii) Excluding cases of cardiovascular disease and cancer incident within a year of baseline. (iii) Adjusting for additional covariates including educational level (≤9, 10–12 and ≥13 years of education), alcohol consumption (grams per week categorized as ‘never’ and then, among consumers, in tertiles as ‘low’, ‘medium’ and ‘high’ intake), physical activity (‘0–2 h of light activity per week’, ‘>2 h of light activity per week’, ‘1–2 h of exercise per week’ and ‘>2 h of exercise per week’) in the subset of studies where this information was available. (iv) Restricting follow-up time to a maximum of 10 years to address possible attenuation by increasing exposure misclassification during follow-up. (v) Excluding the two cohorts that did not report former snus use and where current non-users were classified as never-users, to evaluate possible misclassification bias.^20^^,^^23^ (vi) Adjusting for calendar year at inclusion to address potential variation in diagnosis coding, quality of health care and other background factors over time.

## Results

The 169 103 men constituting the analytical sample yielded 2 857 312 person-years of observation, during which a total of 10 928 deaths occurred. The mean age at entry was 36 years (range 18–99). In the analytical sample, 73.5% had never used any tobacco and 23% were exclusive current snus users. In cohorts reporting former use,^19^^,^^21^^,^^22^^,^^24–26^ 4.8% were exclusive former snus users.

Compared with never-users of tobacco, exclusive current snus users had an increased risk of all-cause mortality (aHR 1.28, 95% CI 1.20–1.35) (Table 3). This increase was mainly driven by excess risks of deaths due to cardiovascular disease (aHR 1.27, 95% CI 1.15–1.41) and causes other than CVD and cancer (aHR 1.37, 95% CI 1.24–1.52). There was also an association with cancer deaths (aHR 1.12, 95% CI 1.00–1.26). Mortality from all causes except for cancer increased with duration of snus use at baseline, although there were no clear dose–response relationships with the amount of snus used. Exclusive former snus users, compared with never-users, had an excess risk of all-cause mortality (aHR 1.15, 95% CI 1.02–1.31) and cancer death (aHR 1.26, 95% CI 1.01–1.57), but apparently not of death from CVD or other causes.

**Table 3:** Pooled aHRs and 95% CIs of death according to cause and snus use at baseline

	Cause of death								
	All causes		Cardiovascular diseases		Cancer		Other causes	
	*n* ^a^	aHR (95% CI)^b^	*n* ^a^	aHR (95% CI)^b^	*n* ^a^	aHR (95% CI)^b^	*n* ^a^	aHR (95% CI)^b^
Tobacco use								
Never-users of tobacco	9272	Ref.	3444	Ref.	2660	Ref.	2098	Ref.
Exclusive current snus users	1410	1.28 (1.20–1.35)	443	1.27 (1.15–1.41)	332	1.12 (1.00–1.26)	511	1.37 (1.24–1.52)
Exclusive former snus users	246	1.15 (1.02–1.31)	83	1.13 (0.91–1.41)	82	1.26 (1.01–1.57)	69	1.14 (0.89–1.45)
Amount (cans/week)^c^								
<4	415	1.28 (1.16–1.41)	166	1.44 (1.23–1.69)	102	1.13 (0.93–1.38)	109	1.24 (1.02–1.51)
4–6	429	1.17 (1.06–1.29)	141	1.18 (1.00–1.40)	116	1.17 (0.96–1.41)	140	1.18 (0.99–1.40)
≥7	528	1.37 (1.25–1.50)	125	1.17 (0.98–1.41)	98	1.01 (0.82–1.24)	253	1.65 (1.43–1.90)
*P* for trend		0.09		0.03		0.22		0.001
Duration, years^c^								
<5	105	1.08 (0.88–1.32)	13	0.98 (0.56–1.72)	12	0.68 (0.38–1.21)	71	1.13 (0.87–1.45)
5–9	189	1.17 (1.00–1.36)	26	0.99 (0.67–1.48)	32	0.94 (0.65–1.35)	114	1.21 (0.99–1.49)
10–14	176	1.31 (1.12–1.52)	38	1.16 (0.84–1.61)	40	1.08 (0.79–1.49)	82	1.46 (1.16–1.84)
≥15	844	1.29 (1.20–1.38)	340	1.32 (1.18–1.48)	215	1.12 (0.97–1.29)	222	1.49 (1.30–1.72)
*P* for trend		0.001		0.001		0.11		0.001

a The numbers of cause-specific deaths do not add up to the total because of missing information regarding the cause of death.

b Adjusted for attained age and BMI.

c Among exclusive current snus users only. Reference category is never-users of tobacco.

Table 4 presents results of sensitivity analyses. The exclusion of the Construction Workers Cohort^19^ inflated aHRs for cardiovascular and cancer mortality. In particular, the aHR of cancer mortality was more pronounced (aHR 1.52, 95% CI 1.15–1.99) among current snus users in the restricted sample. Further adjustment for additional potential confounders (education, alcohol consumption and physical activity) in an additionally restricted sample (excluding the Construction Workers Cohort and the Malmö Diet and Cancer Study) yielded similar results. Current snus use was not associated with cardiovascular mortality after the full sample was restricted to 10 years of follow-up (aHR 1.13, 95% CI 0.93–1.38), whereas cancer and other cause mortality was similar, or more augmented, compared with findings from the main analyses. Lastly, excluding cohorts that did not report former snus use, and additionally adjusting for calendar year, changed the estimates of associations only marginally.

**Table 4:** Pooled aHRs and 95% CIs of death according to cause and current snus use at baseline from sensitivity analyses

Sensitivity analysis	Cause of death^a^	Never-users of tobacco (*n*)	Exclusive current snus users (*n*)	Comparison of snus users and never-users of tobacco [aHR (95% CI)]
Excluding Construction Workers Cohort (CWC)	All causes	3156	177	1.42 (1.21–1.66)
	CVD	1139	56	1.39 (1.05–1.84)
	Cancer	894	60	1.52 (1.15–1.99)
	Other causes	585	43	1.34 (0.97–1.86)
Excluding major CVD and cancer incidents within a year from baseline	All causes	8711	1370	1.28 (1.20–1.36)
	CVD	3253	428	1.27 (1.14–1.40)
	Cancer	2385	310	1.12 (0.99–1.27)
	Other causes	2022	509	1.38 (1.24–1.53)
Excluding the cohorts that do not have information on former snus use	All causes	8396	1389	1.28 (1.21–1.36)
	CVD	3137	439	1.29 (1.16–1.42)
	Cancer	2543	328	1.12 (1.00–1.26)
	Other causes	2093	511	1.38 (1.24–1.52)
Additional adjustment, excluding cohorts lacking covariate information^b^	All causes	2345	158	1.46 (1.22–1.75)
	CVD	846	52	1.56 (1.14–2.14)
	Cancer	787	56	1.51 (1.12–2.03)
	Other causes	585	43	1.20 (0.83–1.72)
Restricting follow-up time to maximum 10 years	All causes	3155	487	1.34 (1.21–1.48)
	CVD	1181	114	1.13 (0.93–1.38)
	Cancer	1001	116	1.24 (1.02–1.52)
	Other causes	825	254	1.57 (1.35–1.83)
Adjusting for calendar year	All causes	9272	1410	1.28 (1.21–1.36)
	CVD	3444	443	1.28 (1.16–1.42)
	Cancer	2660	332	1.13 (1.01–1.28)
	Other causes	2098	511	1.40 (1.26–1.55)

a The numbers of cause-specific deaths do not add up to the total because of missing information regarding the cause of death.

b Excluding CWC and the Malmö Diet and Cancer Study, adjusted for education level, alcohol consumption and physical activity in addition to attained age and BMI.

## Discussion

In this pooled analysis of individual data from eight cohort studies, snus use was associated with an increased risk of death. The excess risk was found for all-cause and cause-specific mortality and was seemingly most attributable to cardiovascular and non-cancer causes. The risk increased in a dose-dependent manner with the baseline reports of duration, but not with amount of snus use.

Our findings are in line with two previous studies on association between snus use and increased risk of death.^17^^,^^18^ In 1994, Bolinder *et al.*^18^ reported an excess overall mortality of 40% (OR 1.4, 95% CI 1.3–1.8) among exclusive current snus users from a 12-year follow up of the Construction Workers Cohort. Another cohort study by Roosaar *et al.*^17^ demonstrated a risk increase of 23% (OR 1.23, 95% CI 1.09–1.40) for exclusive ever snus use (not differentiating between current and former use). Although these studies addressed several potential confounding factors, such as age, area of residence and certain pre-existing conditions, they were unable to adjust for established risk factors such as BMI,^17^ alcohol consumption^18^ and socio-economic status.^17^^,^^18^ As it is known that snus use is associated with higher BMI, increased risk of alcohol abuse and shorter education compared with non-users of tobacco,^28^^,^^30^^,^^34^ lack of adjustment for these factors may generate misleading conclusions. Interestingly, our study supports the results of increased all-cause mortality, even after controlling for these and other confounders.

Cardiovascular diseases and cancer diseases are the two most common causes of death in the Western world.^35^ In our study, we found an association between current snus use and cardiovascular mortality that generally remained after sensitivity analyses, exhibiting a dose–response relationship with duration. However, the association was attenuated after restriction to 10-year follow-up time. One reason for this may be that snus users were young at baseline, on average less than 30 years old. Deaths from cardiovascular diseases at a young age are heavily influenced by genetic factors^36^^,^^37^ rather than lifestyle factors and, therefore, 10 years follow-up time in early adulthood may be too short to evaluate the possible long-term effects of snus use on cardiovascular risk. Nevertheless, due to inconsistency in the results, the increased cardiovascular mortality should be interpreted with caution.

The studies by Bolinder *et al.* and Roosaar *et al.* also showed evidence in support of an increased risk of cardiovascular mortality in snus users.^17^^,^^18^ Moreover, as we have previously reported from this pooling project, although no relationships with incidence could be seen, snus users exhibited increased short-term fatality rates in both stroke and myocardial infarction.^38^^,^^39^ Furthermore, snus discontinuation after diagnosis has been shown to improve myocardial infarction survival.^40^ Snus use has also recently been linked to a higher risk of heart failure.^14^ The current study provides further evidence that snus use has an impact on cardiovascular health. Although the mechanisms behind a possible increase in cardiovascular mortality are still unclear, previous research provides interesting hypotheses: that nicotine may increase endothelial dysfunction^7^^,^^41^ and induce arrhythmia.^42^ Both these processes are important for pathogenesis and mortality in cardiovascular events.^43^^,^^44^

We found a slightly increased risk for death from cancer diseases among snus users. This association did not show any dose–response relationship, but was supported by results of the sensitivity analyses. The increased cancer mortality may reflect poorer survival after cancer diagnosis for snus users, as has been shown for both overall and prostate cancer mortality.^45^^,^^46^ It may also be caused by an increase in particularly lethal cancers among snus users. For example, there is evidence that snus users have increased risk of oesophageal^6^^,^^15^ and rectal^16^ cancer, two diseases with poor survival rates.^47^ However, oesophageal and rectal cancer constitute only a minor part of all cancer diagnoses, which may restrict the impact on overall cancer mortality. Possible mechanisms for increased cancer mortality rates among snus users include nicotine-promoted tumour progression and interaction with antitumor treatment.^9^

Our third category in the cause-specific mortality analyses, ‘other causes of death’, included all deaths that were not classified as ‘cardiovascular deaths’ or ‘cancer deaths’. We found that snus users have an increased risk of other cause death of 37%, exhibiting dose–response trends for both amount and duration of snus use. Our sensitivity analyses were in support of increased other cause death risk.

Self-harm and violence are two common reasons for ‘other cause death’ among Swedish middle-aged men.^48^ As these,^49^^,^^50^ and also snus use,^30^ are associated with alcohol abuse, one could hypothesize that snus use might lead to increased death rates due to self-harm and violence via the association with alcohol abuse. Another possible reason for increased other cause death may be risk taking behaviour among snus users, leading to higher fatality rates from *e.g*. traffic accidents. We consider these topics relevant for further study.

One weakness in our study is that, although we were able to adjust for several of the most important confounders, we cannot fully rule out residual confounding from uncontrolled differences between snus users and non-tobacco users. Furthermore, exposure was only measured at baseline, entailing risk of exposure misclassification, which may partially explain the lack of any clear dose–response trends with increasing amount of snus use in our study. However, previous research has stated that snus use is a fairly stable habit,^51^ and our sensitivity analyses indicate that the misclassification bias introduced by this single baseline measurement is negligible. Another weakness is that former snus use was not measured in two studies. In these, non-current use was defined as never-use, and hence the group ‘never-users of tobacco’ includes a number of former snus users, introducing risk of bias. A sensitivity analysis that excluded these two cohorts showed results similar to those obtained from the main analysis, thus indicating that this bias was minimal.

Our study also has several strengths, the most obvious being its size—there is no larger dataset for investigating the impact of snus use on health. In comparison with previous research, the current study population is more heterogeneous and we had greater possibilities for important adjustments. We were also able to perform valuable sensitivity analyses, reducing the risk of bias from several limitations, and analyses on former snus users, demonstrating overall slightly increased mortality risks, in support of our main results.

In conclusion, our results support an association between snus use and mortality including death from CVD, cancer and other causes. These associations, if causal, are of public health relevance for countries with a high prevalence of snus use. They may also help improve understanding of health effects of other smokeless tobacco and nicotine products.

## Funding

This work was supported by Department of Public Health Sciences, Karolinska Institutet; Region Västerbotten [grant numbers RV-577951, RV-678621]; Region Västernorrland [grant numbers LVNFOU635771, LVNFOU726781, LVNFOU822301]; The Joint Committee of County Councils in Northern Sweden (Visare Norr) [grant numbers VISARENORR542181, VISARENORR646971]; The regional agreement between Umeå University and Region Västerbotten (ALF) [grant number RV-642381]; and The Swedish Society of Medicine [grant number SLS-496881]. The researchers were independent of these funders, and the funders had no role in the study design, data collection, analysis or interpretation, report writing or decision to submit the article for publication.

## References

[1] Holm H, Jarvis MJ, Russell MA, Feyerabend C. Nicotine intake and dependence in Swedish snuff takers. Psychopharmacology (Berl) 1992;108:507–11. 1410167 10.1007/BF02247429

[2] Statistics Sweden. *Tobacco Habits by Indicator, Age and Sex. Percentage and Estimated Numbers in Thousands. Year 2008–2009 - 2016–2017* 2018 http://www.statistikdatabasen.scb.se/pxweb/sv/ssd/START__LE__LE0101__LE0101H/LE0101H25/? rxid=510540eb-a47e-4a02-9d0b-f1fb6bf03b44. (27 November 2018, date last accessed).

[3] Lund I, Lund KE. How has the availability of snus influenced cigarette smoking in Norway? Int J Environ Res Public Health 2014;11:11705–17. 25402565 10.3390/ijerph111111705 PMC4245639

[4] Federal Trade Commission. Federal Trade Commission Smokeless Tobacco Report for 2011. Washington DC: Federal Trade Commission, 2015.

[5] Chang JT, Levy DT, Meza R. Trends and factors related to smokeless tobacco use in the United States. Nicotine Tob Res 2016;18:1740–48. 26995793 10.1093/ntr/ntw090 PMC4941602

[6] International Agency for Research on Cancer. Smokeless Tobacco and Some Tobacco-Specific N-Nitrosamines. Lyon: IARC, 2007.

[7] Adamopoulos D, van de Borne P, Argacha JF. New insights into the sympathetic, endothelial and coronary effects of nicotine. Clin Exp Pharmacol Physiol 2008;35:458–63. 18307741 10.1111/j.1440-1681.2008.04896.x

[8] Heeschen C, Chang E, Aicher A, Cooke JP. Endothelial progenitor cells participate in nicotine-mediated angiogenesis. J Am Coll Cardiol 2006;48:2553–60. 17174197 10.1016/j.jacc.2006.07.066

[9] Heeschen C, Jang JJ, Weis M et al Nicotine stimulates angiogenesis and promotes tumor growth and atherosclerosis. Nat Med 2001;7:833–39. 11433349 10.1038/89961

[10] Hirsch JM, Hedner J, Wernstedt L, Lundberg J, Hedner T. Hemodynamic effects of the use of oral snuff. Clin Pharmacol Ther 1992;52:394–401. 1424411 10.1038/clpt.1992.161

[11] Norberg M, Stenlund H, Lindahl B, Boman K, Weinehall L. Contribution of Swedish moist snuff to the metabolic syndrome: a wolf in sheep's clothing? Scand J Public Health 2006;34:576–83. 17132590 10.1080/14034940600665143

[12] Hansson J, Galanti MR, Magnusson C, Hergens MP. Weight gain and incident obesity among male snus users. BMC Public Health 2011;11:371. 21605406 10.1186/1471-2458-11-371 PMC3118245

[13] Carlsson S, Andersson T, Araghi M et al Smokeless tobacco (snus) is associated with an increased risk of type 2 diabetes: results from five pooled cohorts. J Intern Med 2017;281:398–406. 28164394 10.1111/joim.12592

[14] Arefalk G, Hergens MP, Ingelsson E et al Smokeless tobacco (snus) and risk of heart failure: results from two Swedish cohorts. Eur J Prev Cardiolog 2012;19:1120–27. 10.1177/1741826711420003 21828223

[15] Boffetta P, Hecht S, Gray N, Gupta P, Straif K. Smokeless tobacco and cancer. Lancet Oncol 2008;9:667–75. 18598931 10.1016/S1470-2045(08)70173-6

[16] Araghi M, Galanti MR, Lundberg M et al Smokeless tobacco (snus) use and colorectal cancer incidence and survival: results from nine pooled cohorts. Scand J Public Health 2017;45:741–8. 28994648 10.1177/1403494817714191

[17] Roosaar A, Johansson AL, Sandborgh-Englund G, Axell T, Nyren O. Cancer and mortality among users and nonusers of snus. Int J Cancer 2008;123:168–73. 18412245 10.1002/ijc.23469

[18] Bolinder G, Alfredsson L, Englund A, de Faire U. Smokeless tobacco use and increased cardiovascular mortality among Swedish construction workers. Am J Public Health 1994;84:399–404. 8129055 10.2105/ajph.84.3.399 PMC1614817

[19] Hergens MP, Alfredsson L, Bolinder G, Lambe M, Pershagen G, Ye W. Long-term use of Swedish moist snuff and the risk of myocardial infarction amongst men. J Intern Med 2007;262:351–59. 17697156 10.1111/j.1365-2796.2007.01816.x

[20] Manjer J, Carlsson S, Elmstahl S et al The Malmo Diet and Cancer Study: representativity, cancer incidence and mortality in participants and non-participants. Eur J Cancer Prev 2001;10:489–99. 11916347 10.1097/00008469-200112000-00003

[21] Eriksson M, Holmgren L, Janlert U et al Large improvements in major cardiovascular risk factors in the population of northern Sweden: the MONICA study 1986-2009. J Intern Med 2011;269:219–31. 21158982 10.1111/j.1365-2796.2010.02312.x

[22] Bellocco R, Jia C, Ye W, Lagerros YT. Effects of physical activity, body mass index, waist-to-hip ratio and waist circumference on total mortality risk in the Swedish National March Cohort. Eur J Epidemiol 2010;25:777–88. 20730597 10.1007/s10654-010-9497-6

[23] Carlsson F, Merlo J, Lindstrom M, Ostergren PO, Lithman T. Representativity of a postal public health questionnaire survey in Sweden, with special reference to ethnic differences in participation. Scand J Public Health 2006;34:132–39. 16581705 10.1080/14034940510032284

[24] Pedersen NL, Lichtenstein P, Svedberg P. The Swedish Twin Registry in the third millennium. Twin Res 2002;5:427–32. 12537870 10.1375/136905202320906219

[25] Svensson AC, Fredlund P, Laflamme L et al Cohort profile: the Stockholm Public Health Cohort. Int J Epidemiol 2013;42:1263–72. 23042793 10.1093/ije/dys126

[26] Alfredsson L, Hammar N, Fransson E et al Job strain and major risk factors for coronary heart disease among employed males and females in a Swedish study on work, lipids and fibrinogen. Scand J Work Environ Health 2002;28:238–48. 12199425 10.5271/sjweh.671

[27] Johansson LA, Bjorkenstam C, Westerling R. Unexplained differences between hospital and mortality data indicated mistakes in death certification: an investigation of 1,094 deaths in Sweden during 1995. J Clin Epidemiol 2009;62:1202–9. 19364635 10.1016/j.jclinepi.2009.01.010

[28] Norberg M, Malmberg G, Ng N, Brostrom G. Who is using snus?—Time trends, socioeconomic and geographic characteristics of snus users in the ageing Swedish population. BMC Public Health 2011;11:929. 22169061 10.1186/1471-2458-11-929 PMC3267833

[29] d’Errico A, Ricceri F, Stringhini S et al; LIFEPATH Consortium. Socioeconomic indicators in epidemiologic research: a practical example from the LIFEPATH study. PLoS One 2017;12:e0178071. 28557991 10.1371/journal.pone.0178071 PMC5448763

[30] Norberg M, Malmberg G, Ng N, Brostrom G. Use of moist smokeless tobacco (snus) and the risk of development of alcohol dependence: a cohort study in a middle-aged population in Sweden. Drug Alcohol Depend 2015;149:151–57. 25707707 10.1016/j.drugalcdep.2015.01.042

[31] Di Castelnuovo A, Costanzo S, Bagnardi V, Donati MB, Iacoviello L, de GG. Alcohol dosing and total mortality in men and women: an updated meta-analysis of 34 prospective studies. Arch Intern Med 2006;166:2437–45. 17159008 10.1001/archinte.166.22.2437

[32] Samitz G, Egger M, Zwahlen M. Domains of physical activity and all-cause mortality: systematic review and dose-response meta-analysis of cohort studies. Int J Epidemiol 2011;40:1382–400. 22039197 10.1093/ije/dyr112

[33] Fine JP, Gray RJ. A proportional hazards model for the subdistribution of a competing risk. J Am Stat Assoc 1999;94:496–509.

[34] Engstrom K, Magnusson C, Galanti MR. Socio-demographic, lifestyle and health characteristics among snus users and dual tobacco users in Stockholm County, Sweden. BMC Public Health 2010;10:619. 20955584 10.1186/1471-2458-10-619 PMC2976748

[35] World Health Organization. The Top Ten Causes of Death. 2007 http://www.who.int/mediacentre/factsheets/fs310.pdf (May 2020, date last accessed).

[36] Roberts R, Stewart AF, Wells GA, Williams KA, Kavaslar N, McPherson R. Identifying genes for coronary artery disease: An idea whose time has come. Can J Cardiol 2007;23:7A–15A. 10.1016/s0828-282x(07)71000-0 PMC2787000 17668082

[37] Meyer L, Stubbs B, Fahrenbruch C et al Incidence, causes, and survival trends from cardiovascular-related sudden cardiac arrest in children and young adults 0 to 35 years of age: a 30-year review. Circulation 2012;126:1363–72. 22887927 10.1161/CIRCULATIONAHA.111.076810

[38] Hansson J, Galanti MR, Hergens MP et al Use of snus and acute myocardial infarction: pooled analysis of eight prospective observational studies. Eur J Epidemiol 2012;27:771–79. 22722951 10.1007/s10654-012-9704-8

[39] Hansson J, Galanti MR, Hergens MP et al Snus (Swedish smokeless tobacco) use and risk of stroke: pooled analyses of incidence and survival. J Intern Med 2014;276:87–95. 24548296 10.1111/joim.12219

[40] Arefalk G, Hambraeus K, Lind L, Michaëlsson K, Lindahl B, Sundström J. Discontinuation of smokeless tobacco and mortality risk after myocardial infarction. Circulation 2014;130:325–32. 24958793 10.1161/CIRCULATIONAHA.113.007252

[41] Rohani M, Agewall S. Oral snuff impairs endothelial function in healthy snuff users. J Intern Med 2004;255:379–83. 14871462 10.1046/j.1365-2796.2003.01279.x

[42] Mehta MC, Jain AC, Mehta A, Billie M. Cardiac arrhythmias following intravenous nicotine: experimental study in dogs. J Cardiovasc Pharmacol Ther 1997;2:291–98. 10684470 10.1177/107424849700200407

[43] Egashira K. Clinical importance of endothelial function in arteriosclerosis and ischemic heart disease. Circ J 2002;66:529–33. 12074266 10.1253/circj.66.529

[44] Henkel DM, Witt BJ, Gersh BJ et al Ventricular arrhythmias after acute myocardial infarction: a 20-year community study. Am Heart J 2006;151:806–12. 16569539 10.1016/j.ahj.2005.05.015

[45] Nordenvall C, Nilsson PJ, Ye W, Andersson TM, Nyren O. Tobacco use and cancer survival: a cohort study of 40,230 Swedish male construction workers with incident cancer. Int J Cancer 2013;132:155–61. 22492255 10.1002/ijc.27587

[46] Wilson KM, Markt SC, Fang F et al Snus use, smoking and survival among prostate cancer patients. Int J Cancer 2016;139:2753–59. 27582277 10.1002/ijc.30411 PMC5061636

[47] Bergman O, Fredholm L, Hont G et al Cancer i Siffror 2018. Stockholm: Socialstyrelsen and Cancerfonden, 2018.

[48] Statistics Sweden. *Statistical Database, Cause of Death* 2020 https://sdb.socialstyrelsen.se/if_dor/val_eng.aspx (24 June 2020, date last accessed).

[49] Vinson DC, Maclure M, Reidinger C, Smith GS. A population-based case-crossover and case-control study of alcohol and the risk of injury. J Stud Alcohol 2003;64:358–66. 12817824 10.15288/jsa.2003.64.358

[50] Britton A, McPherson K. Mortality in England and Wales attributable to current alcohol consumption. J Epidemiol Community Health 2001;55:383–88. 11350993 10.1136/jech.55.6.383 PMC1731912

[51] Norberg M, Lundqvist G, Nilsson M, Gilljam H, Weinehall L. Changing patterns of tobacco use in a middle-aged population: the role of snus, gender, age, and education. Glob Health Action 2011 ;4: 5613. doi: 10.3402/gha.v4i0.5613. 10.3402/gha.v4i0.5613 PMC3118776 21695071

